# The complete chloroplast genome sequence of *Populus szechuanica*, a medicinal plant for child anesthesia application

**DOI:** 10.1080/23802359.2021.1882907

**Published:** 2021-03-01

**Authors:** Xun Chen, Ce-Hua Ou

**Affiliations:** Department of Pain Management, the Affiliated Hospital of Southwest Medical University, Luzhou, China

**Keywords:** *Populus szechuanica*, chloroplast genome, phylogenetic analysis, child anesthesia application

## Abstract

*Populus szechuanica* C.K. Schneid. is used for wind and sand fixation, farmland protection, soil and water conservation. And some varieties bud fat and inflorescence are available for medicinal use; poplar bark contains tannin and can be used as tanning material. It is also widely used in afforestation and greening in cities across China. In addition, the *P. szechuanica* also showed vital important application values on the child anesthesia, which could significantly reduce the occurrence of hypotension after anesthesia. The complete chloroplast genome sequence of *P. szechuanica* was characterized from Illumina pair-end sequencing. The chloroplast genome of *P. szechuanica* was 156,717 bp in length, containing a large single-copy region (LSC) of 84,900 bp, a small single-copy region (SSC) of 16,527 bp, and two inverted repeat (IR) regions of 27,645 bp. The overall GC content is 36.70%, while the corresponding values of the LSC, SSC, and IR regions are 34.5%, 30.7%, and 41.9%, respectively. The genome contains 131 complete genes, including 86 protein-coding genes (68 protein-coding gene species), 37 tRNA genes (29 tRNA species) and 8 rRNA genes (4 rRNA species). The Maximum Likelihood phylogenetic analysis showed that *P. szechuanica* and *P. koreana* clustered together as sisters to other *Populus* species.

## Introduction

The so-called pediatric anesthesia refers to the anesthesia from the neonate to the age of 12 years, but the main target is children under 6 years old, especially the physiological characteristics of children under 3 years old have obvious differences with adults. Many special problems encountered in pediatric anesthesia and their use in the special measures are also based on this age. Pediatrics are not the epitome of adults. The physiological functions of newborns and infants are very different from those of adults. Therefore, children’s anesthesia must be familiar with the anatomical and physiological characteristics of children, and the anesthesia techniques and methods applied to adults must be properly adjusted, improved or processed and calculated accurately before they can be applied to children of different ages and can ensure children with the safety of anesthesia. In our previous study, we have proved that the bud fat of the *Populus szechuanica* C.K. Schneid. has important application values on the cenesthesia, which could significantly reduce the occurrence of hypotension after anesthesia. Thus, in this present research, we further want to explore the relationship between the *Populus szechuani* biological activity and the complete chloroplast genome sequence. The study of the complete chloroplast genome sequence of *Populus szechuani* could provide reveal the related mechanism of the *Populus szechuani* biological activity.

*Populus szechuani* is used for wind and sand fixation, soil and water conservation; It is also used as building materials and fuel wood, furniture, carving crafts; poplar leaf can be used as feed for wild animals and livestock; bud fat can be used as a yellow-brown dye, and some varieties Bud fat and inflorescence are available for medicinal use; poplar bark contains tannin and can be used as tanning material. It is also widely used in afforestation and greening in cities across China. *P. szechuanica* has high ecological and economic value with high levels of intraspecific genetic diversity. *P. szechuanica* has wide geographic distribution, high intraspecific polymorphism, adaptability to different environments, combined with a relatively small genome size. Consequently, *P. szechuanica* represents an excellent model for understanding how different evolutionary forces have sculpted the variation patterns in the genome during the process of population differentiation and ecological speciation (Neale and Antoine 2011). Moreover, we can develop conservation strategies easily when we understand the genetic information of *P. szechuanica*. In the present research, we constructed the whole chloroplast genome of *P. szechuanica* and understood many genome variation information about the species, which will provide beneficial help for population genetics studies of *P. szechuanica.*

The fresh leaves of *P. szechuanica* were collected from Xizang (91°10′E; 29°41′N). Fresh leaves were silica-dried and taken to the laboratory until DNA extraction. The voucher specimen (ZCY001) was laid in the Herbarium of the Affiliated Hospital of Southwest Medical University and the extracted DNA was stored in the −80 °C refrigerator of the Key Laboratory of Department of Pain Management. We extracted total genomic DNA from 25 mg silica-gel-dried leaf using a modified CTAB method (Doyle 1987). The whole-genome sequencing was then conducted by Biodata Biotechnologies Inc. (Hefei, China) with Illumina Hiseq platform. The Illumina HiSeq 2000 platform (Illumina,San Diego, CA) was used to perform the genome sequence. We used the software MITObim 1.8 (Hahn et al. 2013) and metaSPAdes (Nurk et al. 2017) to assemble chloroplast genomes. We used *Populus qamdoensis* (GenBank: NC040868) as a reference genome. We annotated the chloroplast genome with the software DOGMA (Wyman et al. 2004), and then corrected the results using Geneious 8.0.2 (Campos et al. 2016) and Sequin 15.50 (http://www.ncbi.nlm.nih.gov/Sequin/).

The complete chloroplast genome of *P. szechuanica* (GenBank accession number MT593369) was characterized from Illumina pair-end sequencing. The chloroplast genome of *Populus szechuanica* was 156,717 bp in length, containing a large single-copy region (LSC) of 84,900 bp, a small single-copy region (SSC) of 16,527 bp, and two inverted repeat (IR) regions of 27,645 bp. The overall GC content is 36.70%, while the corresponding values of the LSC, SSC, and IR regions are 34.5%, 30.7%, and 41.9%, respectively. The genome contains 131 complete genes, including 86 protein-coding genes (68 protein-coding gene species), 37 tRNA genes (29 tRNA species) and 8 rRNA genes (4 rRNA species).

**Figure 1.**
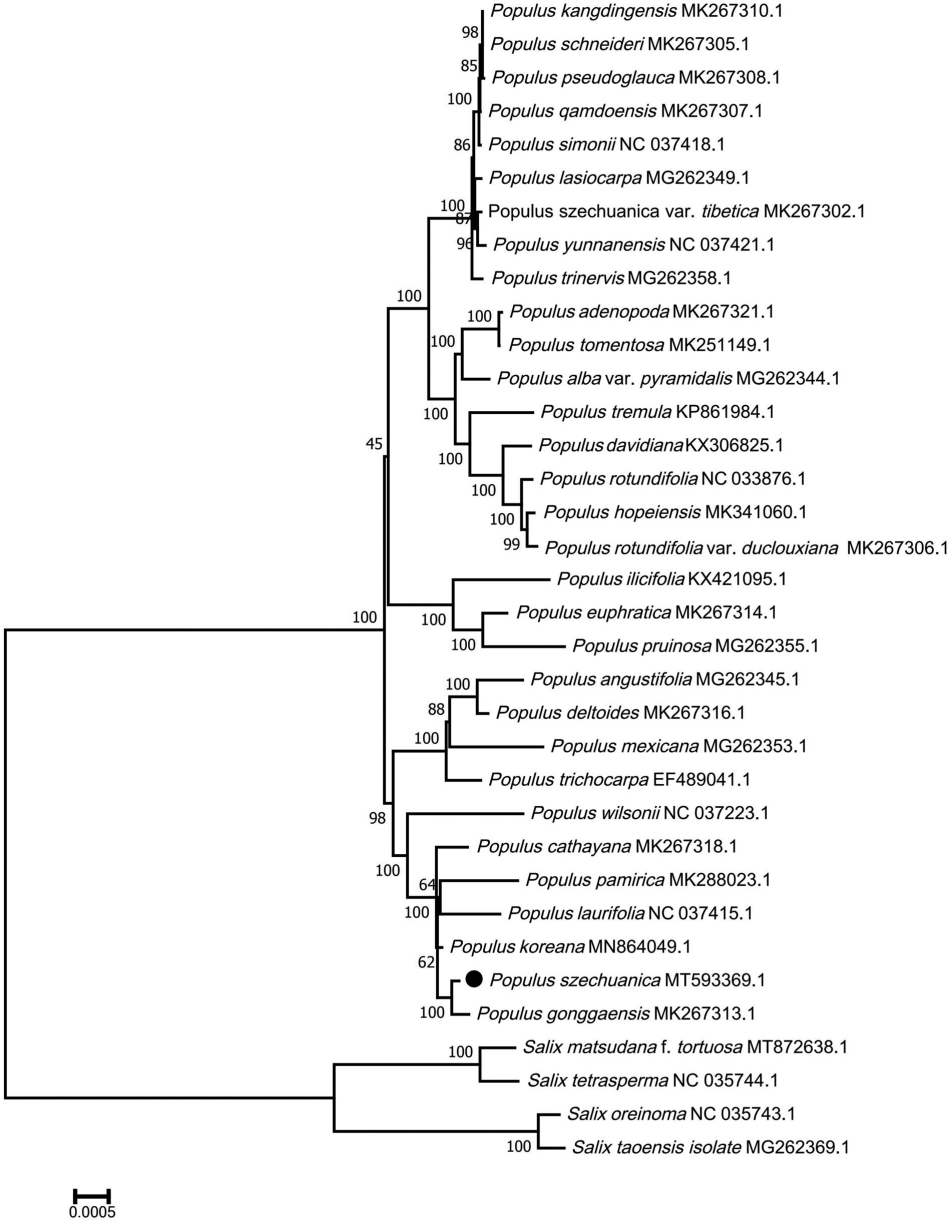
Maximum Likelihood (ML) analysis of *Populus szechuanica* and other related species based on the complete chloroplast genome sequence.

We used the complete chloroplast genomes sequence of *P. szechuanica* and 33 other related species to construct phylogenetic tree. The 34 chloroplast genome sequences were aligned with MAFFT (Katoh and Standley 2013), and then the Maximum Likelihood (ML) tree was constructed by MEGA 7.0 (Kumar et al. 2016). The ML phylogenetic analysis showed that *P. szechuanica* and *P. gonggaensis* have the closest genetic relationship (Figure 1).

## Data Availability

The genome sequence data that support the findings of this study are openly available in GenBank of NCBI at (https://www.ncbi.nlm.nih.gov/) under the accession no. MT593369. The associated BioProject, SRA, and Bio-Sample numbers are PRJNA682691, SRR13203218, and SAMN17005496 respectively.
